# Isolation of Terbinafine-Resistant *Trichophyton rubrum* from Onychomycosis Patients Who Failed Treatment at an Academic Center in New York, United States

**DOI:** 10.3390/jof9070710

**Published:** 2023-06-28

**Authors:** Jonathan K. Hwang, Wayne L. Bakotic, Jeremy A. W. Gold, Cynthia M. Magro, Shari R. Lipner

**Affiliations:** 1 Weill Cornell Medicine, New York, NY 10021, USA; jkh4001@med.cornell.edu (J.K.H.); cym2003@med.cornell.edu (C.M.M.); 2 Bako Diagnostics, Alpharetta, GA 30005, USA; wbakotic@bakodx.com; 3 Centers for Disease Control and Prevention, Atlanta, GA 30329, USA; leo3@cdc.gov

**Keywords:** nails, nail diseases, antifungal resistance, antimicrobials, antimicrobial resistance

## Abstract

Onychomycosis is a common nail infection. Terbinafine-resistant dermatophyte infections pose an emerging global public health concern, but few cases have been described in the United States. We retrospectively reviewed and characterized clinical, histopathological, and mycological features of patients with mycologically confirmed onychomycosis who failed oral terbinafine treatment for onychomycosis at a U.S. academic nail referral center and ascertained for terbinafine-resistant isolates. During 1 June 2022–31 January 2023 at Weill Cornell Medicine in New York City, USA, 96 patients with mycologically confirmed onychomycosis were treated with oral terbinafine. Among 64 patients with adequate follow-up, 36 had clinical or complete cure. Of 28 patients who failed treatment, 17 underwent terbinafine resistance testing. *Trichophyton rubrum* with terbinafine resistance-conferring mutations was isolated from two patients. Overall, terbinafine failures for onychomycosis were relatively common, with some cases associated with terbinafine-resistant *T. rubrum* infections. These findings underscore the need for a clinical awareness of this emerging problem and public health efforts to monitor and prevent spread. We highlight the importance of diagnostic testing and species identification for onychomycosis patients and the increasingly important role of fungal identification and susceptibility testing to guide therapy.

## 1. Introduction

Onychomycosis, a fungal nail infection, is the most common nail disorder encountered in clinical practice, with a worldwide prevalence of 5.5% [1]. Although sometimes dismissed as a purely cosmetic concern, it may cause pain and psychosocial distress, and predisposes diabetic patients to life-threatening bacterial superinfections [1]. The most frequent causative organisms are dermatophytes (70%), most often *Trichophyton rubrum* and *Trichophyton interidigitale*, followed by non-dermatophyte molds (NDM) (e.g., *Aspergillus*, *Fusarium*) and yeasts (e.g., *Candida*) (30%) [2]. The primary treatment for onychomycosis is terbinafine, an oral antifungal with mycologic cure rates of 70–79% and complete cure rates of 38–59% [3]. 

Terbinafine-resistant dermatophytoses have become an emerging public health concern, reaching epidemic proportions in India, with cases also identified in Asia, Europe, and North America [4,5,6,7,8]. Resistance is primarily caused by point mutations in the squalene epoxidase (SQLE) gene, encoding terbinafine’s primary target [9]. In the United States (U.S.), where most patients with suspected onychomycosis do not receive mycologic testing, terbinafine resistance testing is rarely performed. The prevalence and characteristics of terbinafine-resistant dermatophyte infections, particularly onychomycosis, are thus not well-described [1,2]. Recently, mutations associated with terbinafine resistance have been increasingly identified in US onychomycosis patients [9]. The continual monitoring for and characterizing features of these terbinafine-resistant onychomycosis patients are important to inform clinical practice and public health prevention measures. 

## 2. Materials and Methods

During 1 June 2022–31 January 2023, patients who received terbinafine for onychomycosis, confirmed by presence of hyphae on histopathology using periodic acid–Schiff (PAS) staining of nail clippings at Weill Cornell Dermatology (New York, NY, USA) were identified. Patients who failed therapy, defined as lack of clinical or complete cure following a standard (3-month) terbinafine treatment regimen (250 mg/day), and presented for subsequent follow-up, were selected for terbinafine resistance testing. A retrospective chart review was performed to identify patient demographics, underlying conditions, clinical features, and treatment history. Repeat nail clippings were collected for PAS staining and histopathologic analysis by a Weill Cornell Medicine dermatopathologist (CMM). Nail samples were further analyzed by Bako Diagnostics Laboratory (Alpharetta, GA, USA) using polymerase chain reaction (PCR) molecular assay, fungal culture, and terbinafine resistance reflex testing with a real-time PCR assay that detects 12 terbinafine resistance-conferring mutations in the SQLE gene (Figure S1, Table S1) [9]. DNA extraction from nail samples was performed using Hamilton Microlab STAR systems with the Omega plant DNA extraction kit [9].

## 3. Results

During 1 June 2022–31 January 2023, 96 patients with mycologically confirmed onychomycosis received oral terbinafine therapy. Of the 64 patients presenting for follow-up after a 3-month treatment course, 36 (56%) had clinical or complete cure. Among these, 17 presented for subsequent follow-up and underwent terbinafine resistance testing; most were male (*n* = 12), aged > 50 years (median: 54, range 31–76), and non-Hispanic White (*n* = 14) (Table 1). Fourteen had toenail involvement, and four had fingernail involvement (one had both). Eleven patients reported underlying conditions, most frequently hypertension (*n* = 3) and sleep apnea (*n* = 3). Median reported onychomycosis duration was 6 years (range 1–15). Other previous onychomycosis treatments included topical ciclopirox (*n* = 3), oral itraconazole (*n* = 2), oral fluconazole (*n* = 2), and topical efinaconazole (*n* = 1). Pathogens identified by PCR or culture included *Trichophyton rubrum* (*n* = 11), *Fusarium* (*n* = 3), *Aspergillus* (*n* = 2), and *Scytalidium* (*n* = 1) species. Three patients were diagnosed with dermatophytomas on histopathology.

**Figure 1:**
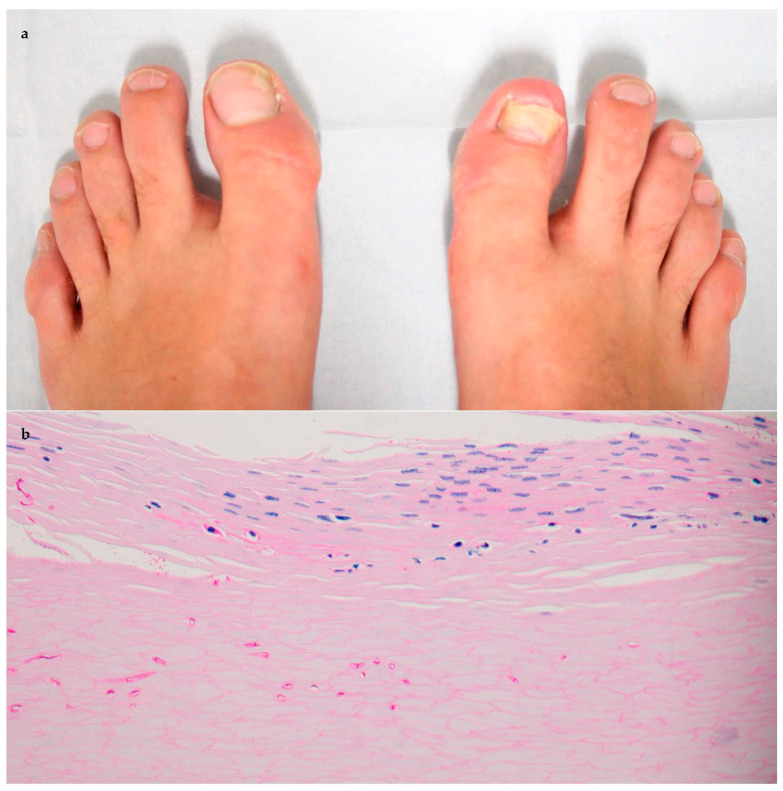
Patient #3 clinically exhibiting (**a**) onycholysis of great toenails with subungual debris, with histopathology with periodic acid–Schiff staining 40 × revealing (**b**) septated hyphal elements coursing through subungual horn and plate.

Terbinafine-resistant *T. rubrum* was isolated from two patients. One patient (patient #2) was a 41-year-old man with a history of melanoma. He had onychomycosis for several years and had previously failed treatment with one course of terbinafine and two courses of fluconazole. From his nail specimen, one mutated *T. rubrum* population was identified (Phe397Leu). His onychomycosis resolved after treatment with a 3-month course of itraconazole (200 mg daily). The other patient (patient #3) was a 72-year-old male with hypertension, atrial fibrillation, benign prostate hyperplasia, and Waldenstrom’s macroglobulinemia. He had onychomycosis for several years and had previously failed treatment with one course of terbinafine, followed by topical efinaconazole (Figure 1). From his nail specimen, three distinct *T. rubrum* populations were identified, each harboring different SQLE mutations (Phe397Leu, Phe415Ser, and Phe415Ile). He died before his follow-up onychomycosis appointment. No terbinafine resistance mutations were identified from the remaining patients, who were treated with topical efinaconazole (*n* = 8), oral terbinafine 250 mg (*n* = 4), or oral fluconazole 150 mg (*n* = 1).

## 4. Discussion

In this study of patients with mycologically confirmed onychomycosis at a major academic nail center, terbinafine treatment failures occurred in nearly half of patients (28/64 of those with adequate post-therapy follow-up). Terbinafine-resistant *T. rubrum* was isolated from 2/17 patients who failed terbinafine therapy. Given the high worldwide prevalence of onychomycosis and the clinical importance of terbinafine as a treatment option, the detection of terbinafine resistance in the U.S. constitutes a public health concern. This issue warrants increased attention and prevention efforts, including strengthened surveillance and antifungal stewardship efforts. Physicians should be aware of terbinafine resistance as a potential cause of onychomycosis treatment failures in patients with mycologically confirmed onychomycosis.

Resistance is due to point mutations causing amino acid substitutions in the SQLE gene, the primary drug target of terbinafine [9]. Subsequent conformational rearrangements in the SQLE protein prevent proper inhibitor binding, causing a reduced susceptibility and an increased minimum inhibitory concentration (MIC) in these strains [10]. Identified point mutations have been primarily isolated to single nucleotide polymorphisms at four amino acid loci: Leu393, Phe397, Phe416, and His440 [9]. The degree of resistance is directly related to the specific amino acid substitution present, with Leu393 and Phe397 variants demonstrating the highest increased MIC [6]. Mutations with Phe397 and/or Phe415 were present in the patients analyzed in our study.

In the context of emerging terbinafine resistance, mycological testing before initiating antifungal treatment is particularly important to avoid antifungal overprescribing, which may drive resistance [5]. Notably, clinical examination alone has poor sensitivity and specificity for onychomycosis diagnosis. Available diagnostic tests include potassium hydroxide (KOH) examination (67–93% sensitivity, 38–78% specificity), fungal culture (31–59% sensitivity, 83–100% specificity), histopathology with PAS (92% sensitivity, 72% specificity), and PCR (95% sensitivity, 100% specificity) [2]. Specific method choice is dictated by patient characteristics, time to initiate therapy, cost, sensitivity and specificity of techniques, and physician expertise. The 2013 Choosing Wisely campaign recommended that onychomycosis confirmatory testing be performed before initiating systemic therapy [11]. However, in a retrospective analysis study of 1774 patients with onychomycosis diagnosis, confirmatory testing decreased by 37% in the period of 2013–2018, compared with 2002–2012 preceding the campaign [12]. A commercial database study of 121,386 U.S. onychomycosis patients during 2018 similarly reported that <10% had received confirmatory testing prior to terbinafine treatment [13]. Our study highlights the use of PCR as a rapid and accurate diagnostic confirmatory test, allowing for both species identification and subsequent resistance testing [1].

Onychomycosis therapy is guided by considering disease severity, the number of nails affected, comorbidities, concomitant medications, causative organism, and susceptibility to antifungal drugs [3]. Oral itraconazole, approved by the U.S. Food and Drug Administration (FDA), and oral fluconazole, approved in other countries and frequently used off-label in the U.S., could be considered for terbinafine-resistant isolates [4]. Although effective, these drugs have several disadvantages compared with terbinafine, including more drug–drug interactions and a greater cost [2]. Notably, some dermatophytoses exhibit resistance to these antifungals, further compounding the public health concern [8]. FDA-approved topical treatments, including ciclopirox, efinaconazole, and tavaborole, have also been used for treating terbinafine-resistant dermatophytosis, as well as combination therapies [2,4]. Newer systemic antifungals generally reserved for invasive fungal disease (e.g., voriconazole, posaconazole) have also been employed, but have not been studied specifically for terbinafine-resistant onychomycosis [4,14].

In addition to the terbinafine-resistant *T. rubrum* isolates identified from two patients, treatment failures in this study could also potentially reflect treatment non-adherence, reinfection, NDM infection, and the presence of dermatophytoma. Patient counseling on treatment adherence and strategies to prevent recurrence (e.g., the avoidance of nail trauma, the treatment of concomitant tinea pedis) might help ensure successful therapy [3]. The treatment of NDM onychomycosis is often challenging, typically requiring combination systemic–topical therapies [15]. The recognition of dermatophytomas, which are characterized clinically by dense white-yellow longitudinal streaks or patches and histopathological evidence of dermatophyte hyphae or fungal elements in a densely compacted mass, generally requiring treatment with topical efinaconazole or tavaborole, also remains important [16].

Study limitations are the small sample size and single-center design at an academic nail referral center, which likely impacted the representativeness of our findings. Determining U.S. epidemiology and the clinical features of terbinafine-resistant onychomycosis is needed to help inform prevention efforts and clinical guidance. Additionally, DNA-based testing, as utilized for our clinical isolates, lacks data on MIC for terbinafine. The terbinafine resistance testing utilized also focused solely on four previously reported hotspots in the SQLE gene. A more thorough MIC surveillance, as well as data on other clinically significant mutation sites, may improve the mapping of resistance development.

## 5. Conclusions

Overall, our study highlights the emergence of terbinafine-resistant *T. rubrum* in a proportion of onychomycosis treatment failures. We emphasize the importance of mycologic testing in clinical practice for suspected onychomycosis, antifungal stewardship to preserve the availability of current treatments, and public health efforts to monitor and control the spread of terbinafine resistance. We also demonstrate the increasing importance of fungal identification and antifungal susceptibility testing in the choice of therapy, especially with treatment failures.

## Figures and Tables

**Table 1:** Demographics, clinical history, physical examination findings, diagnostic tests, and treatments of patients that underwent terbinafine resistance testing.

#	Age/Sex	Race	Past Medical History	Nails Affected	Duration	Prior Treatment(s)	Physical Examination	Repeat Pathology	PCR	Culture	Terbinafine Resistance	Treatment (Outcome)
1	54/F	White	None	R toenails	Multiple years	Itraconazole Terbinafine 250 mg × 3 months	Thickening, yellow discoloration	Not collected	*Trichophyton rubrum*	*Trichophyton rubrum*	Susceptible	Efinaconazole 10%
2	72/M	White	HTN, atrial fibrillation, BPH, Waldenstrom’s macroglobulinemia	All toenails	Multiple years	Terbinafine 250 mg × 3 months	Thickening, yellow discoloration	Not collected	*Trichophyton rubrum*	No fungus isolated	Resistant (3 mutated populations: Phe397Leu, Phe415Ser, Phe415Ile)	Efinaconazole 10%
3	41/M	White	Melanoma	R1/L1 toenails	Multiple years	Terbinafine 250 mg × 3 months 2 × Fluconazole 150 mg × 12 months	Onycholysis, subungual debris	Hyphae	*Trichophyton rubrum*	Not performed	Resistant (1 mutated population: Phe397Leu)	Itraconazole 200 mg × 3 months (improved)
4	45/F	White Hispanic	None	R1 toenail	1 year	Terbinafine 250 mg × 3 months	Yellow streak extending to lunula	Hyphae	*Aspergillus*	*Aspergillus*	Susceptible	Fluconazole 150 mg × 3 months (failed) Efinaconazole 10%
5	56/M	White	Essential tremor, migraines, Sjogren’s, sleep apnea	R1 toenail	Multiple years	Terbinafine 250 mg × 3 months	Onycholysis, yellow discoloration, onycholysis, white patch	Hyphae	*Trichophyton rubrum*	Not performed	Susceptible	Efinaconazole 10%
6	25/M	White	None	All toenails	2 years	Terbinafine × 8 months	Xanthonychia, onycholysis, subungual debris, crumbling	Hyphae	*Trichophyton rubrum*	*Trichophyton rubrum*	Susceptible	Terbinafine 250 mg × 3 months
7	51/M	White	HTN, obesity, sleep apnea	R1/L1 fingernails; R4 toenail	6 years	Terbinafine	Onycholysis, thickening of fingernails, yellow streak of R4 toenail	Dermatophytoma	*Trichophyton rubrum*	*Trichophyton rubrum*	Susceptible	Terbinafine 250 mg × 3 months (improved)
8	53/F	White	GDM, Grave’s disease, prothrombin mutation	L1 toenail	2 years	Terbinafine 250 mg × 3 months	Onychomadesis	Hyphae	*Fusarium*	No fungus isolated	Susceptible	Lost to follow-up
9	31/M	White	IBS, depression, asthma	L toenails	10 years	4 × Terbinafine 250 mg × 3 months	Hyperkeratotic nail bed, subungual debris, yellow discoloration	Dermatophytoma	*Trichophyton rubrum*	No fungus isolated	Susceptible	Efinaconazole 10%
10	60/M	White	None	L1 toenail	10 years	Terbinafine Multiple × Itraconazole Multiple × Fluconazole Efinaconazole 10% × 2 months	Proximal white streak	Hyphae	*Trichophyton rubrum*	No fungus isolated	Susceptible	Efinaconazole 10%
11	76/M	White Hispanic	HTN, CAD, DM, obesity, BPH, PUD, aortic dissection, kidney stones, sleep apnea, arrhythmia	R1 fingernail	15 years	Terbinafine 250 mg × 6 weeks	Thickening, onycholysis, subungual debris	Not collected	*Scytalidum,* *Candida guilliermondii*	*Scytalidium dimidiatum*	Susceptible	Efinaconazole 10%
12	37/M	White	None	R3 fingernail	Multiple years	2 × Terbinafine 250 mg × 2 months Ciclopirox 8% × 1 year	Severe onycholysis, hyperkeratotic nail bed, nail fold fluctuance without tenderness	Hyphae	*Fusarium*	*Fusarium*	Susceptible	Efinaconazole 10%
13	68/M	White	Raynaud’s	All toenails	Multiple years	Multiple × Terbinafine	Xanthonychia, onycholysis, subungual debris	Hyphae	*Trichophyton rubrum*	*Fusarium, Trichophyton rubrum*	Susceptible	Efinaconazole 10%
14	61/M	White	Kidney stones, allergic rhinitis	R1/R2 toenails	10 years	Terbinafine	Xanthonychia, thickening, subungual debris	Hyphae	*Trichophyton rubrum*	No fungus isolated	Susceptible	Terbinafine 250 mg × 3 months
15	68/M	Asian	HTN	R1/L1 fingernails	5 years	Terbinafine × 6 weeks Ciclopirox × 7 months	Onycholysis	Hyphae	*Trichophyton rubrum*	No fungus isolated	Susceptible	Terbinafine 250 mg × 6 weeks
16	63/F	White	Osteoporosis	L1 toenail	2 years	Ciclopirox × 1 month Terbinafine × 3 months	Severe onycholysis, subungual debris	Hyphae	*Aspergillus*	*Aspergillus*	Susceptible	Lost to follow-up
17	31/F	White	None	L1 toenail	3 years	Terbinafine × 3 months	Severe onycholysis	Dermatophytoma	*Fusarium*	*Fusarium*	Susceptible	Efinaconazole 10%

Abbreviations: PCR: polymerase chain reaction testing, HTN: hypertension, BPH: benign prostate hyperplasia, GDM: gestational diabetes mellitus, IBS: irritable bowel syndrome, CAD: coronary artery disease, DM: diabetes mellitus, PUD: peptic ulcer disease.

## Data Availability

All data generated or analyzed during this study are included in this article. Further inquiries can be directed to the corresponding author.

## Supplementary Materials

The following supporting information can be downloaded at: https://www.mdpi.com/article/10.3390/jof9070710/s1, Figure S1: Mutation region within the squalene epoxidase gene of *Trichophyton rubrum*; Table S1: List of mutations (single nucleotide polymorphisms) and their non-synonymous changes in the amino acid.
