# Supplementary material for: Isolation of Terbinafine-Resistant Trichophyton rubrum from Onychomycosis Patients Who Failed Treatment at an Academic Center in New York, United States
Source: J Fungi (Basel). 2023 Jun 28;9(7):710. doi: 10.3390/jof9070710 (PMC10381657; doi:10.3390/jof9070710)
Supplement: Supplementary file 1 [file jof-09-00710-s001.zip › jof-2465431-supplementary.pdf]

Supplementary Files

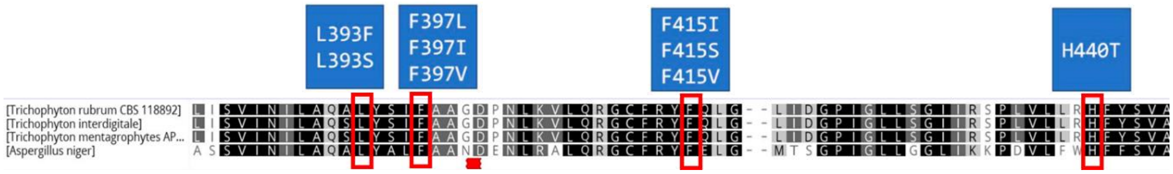

Figure S1. Mutation region within the squalene epoxidase gene of *Trichophyton rubrum*.

Gene position (bp)	Wild type	Point Mutations
1177-1179	**TTA** (Leu393)	**TTT** (Leu393Phe)
		**TCA** (Leu393Ser)
		**TTC** (Leu393Phe)
1189-1191	**TTC** (Phe397)	**TTA** (Phe397Leu)
		**CTC** (Phe397Leu)
		**ATC** (Phe397Ile)
		**GTC** (Phe397Val)
		**TTG** (Phe397Leu)
1305-1307	**TTC** (Phe415)	**ATC** (Phe415Ile)
		**GTC** (Phe415Val)
		**TCC** (Phe415Ser)
1380-1382	**CAT** (His440)	**TAT** (His440Tyr)

Table S1. List of mutations (single nucleotide polymorphisms) and their non-synonymous changes in the amino acid
